# icMRCI+Q Study of the Spectroscopic Properties of the 14 Λ-S and 49 Ω States of the SiN^−^ Anion in the Gas Phase

**DOI:** 10.3390/molecules23010210

**Published:** 2018-01-20

**Authors:** Wei Xing, Jinfeng Sun, Deheng Shi, Zunlue Zhu

**Affiliations:** 1 School of Materials Science and Engineering, Henan University of Science and Technology, Luoyang 471023, China; wei19820403@163.com; 2 College of Physics and Electronic Engineering, Xinyang Normal University, Xinyang 464000, China; 3 College of Physics and Material Science, Henan Normal University, Xinxiang 453007, China; scattering@sina.com.cn (D.S.); zl-zhu@htu.edu.cn (Z.Z.)

**Keywords:** spectroscopic parameter, electron affinity, spin-orbit coupling effect, Franck-Condon factor, radiative lifetime

## Abstract

This paper calculates the potential energy curves of the 14 Λ-S and 49 Ω states, which come from the first three dissociation channels of the SiN^−^ anion. These calculations are conducted using the valence internally contracted multireference configuration interaction and the Davidson correction approach. Core-valence correlation and scalar relativistic corrections are taken into account. The potential energies are extrapolated to the complete basis set limit. The spin-orbit coupling is computed using the state interaction approach with the Breit–Pauli Hamiltonian. We found that the X^1^Σ^+^ (*υ*′′ = 0–23) and a^3^Σ^+^ (*υ*′ = 0–2) states of SiN^−^ are stable at the computed adiabatic electron affinity value of 23,262.27 cm^−1^ for SiN. Based on the calculated potential energy curves, the spectroscopic parameters and vibrational levels were determined for all stable and metastable Λ-S and Ω states. The computed adiabatic electron affinity of SiN and the spectroscopic constants of SiN^−^ (X^1^Σ^+^) are all in agreement with the available experimental data. The d^3^Σ^+^, 2^5^Σ^+^, 1^5^Δ, and 1^5^Σ^−^ quasi-bound states caused by avoided crossings were found. Calculations of the transition dipole moment of a^3^Σ^+^_1_ to X^1^Σ^+^_0+_ are shown. Franck-Condon factors, Einstein coefficients, and radiative lifetimes of the transition from the a^3^Σ^+^_1_ (*υ*′ = 0–2) to the X^1^Σ^+^_0+_ state are evaluated.

## 1. Introduction

SiN^−^ is analogous with the known interstellar CN^−^ anion [1]. Several silicon-containing nitrogen chains (SiN, SiCN, and SiNC) have been detected in the interstellar medium [2,3,4]. Consequently, SiN^−^ is a tantalizing potential interstellar anion candidate [5]. Furthermore, the SiN^−^ anion is a compound of considerable importance in gas-phase ion chemistry [6], laser-induced plasmas [7], semiconductor chemistry [8], and microelectronics [9]. However, very few experiments [10] and computations [11,12,13,14,15,16] have been conducted for its spectroscopic properties. Based on the aforementioned facts, we systematically investigated the spectroscopic properties of the SiN^−^ anion using a highly accurate ab initio approach. 

Experimentally, Meloni et al. [10] used anion photoelectron spectroscopy to obtain the SiN (X^2^Σ^+^, A^2^Π) + e^−^ ← SiN^−^ (X^1^Σ^+^) transitions for the first time in 2004. They determined the equilibrium bond length (*R_e_*), harmonic frequency (*ω_e_*), and dissociation energy (*D*_0_) for the X^1^Σ^+^ state of SiN^−^. The adiabatic electron affinity (AEA) of SiN was also determined. 

Theoretically, only six groups of computations [11,12,13,14,15,16] were performed. In 1989, Peterson and Woods [11] used the Møller–Plesset perturbation theory with single, double, and quadruple substitutions (MP4SDQ) and two large Gaussian basis sets to calculate the AEA (26,826.21 cm^−1^) of SiN and the spectroscopic constants of the SiN^−^ (X^1^Σ^+^). In 1992, McLean et al. [12] studied the potential energy curve (PEC) of SiN^−^ (X^1^Σ^+^) at the singles and double excitation from a single reference configuration interaction plus Davidson correction (SDCI + Q) levels of theory. Kalcher [13] in 2002 studied the AEA (23,083.58 cm^−1^) of SiN and the PECs of X^1^Σ^+^ and a^3^Σ^+^ states using the complete active space in conjunction with the averaged coupled pair functional (CAS-ACPF) approach using the cc-pVQZ basis set. In 2003, Midda and Das [14] calculated the spectroscopic constants and molecular properties of X^1^Σ^+^ states using a hybrid HF/DF B3LYP method with four different basis sets. Kerkines and Mavridis [15] in 2005 calculated the spectroscopic constants and energetics for the SiN (X^2^Σ^+^, A^2^Π) and SiN^−^ (X^1^Σ^+^, a^3^Σ^+^) at the level of the theory of the restricted coupled-cluster method with all singles and doubles and noniterative inclusion of triples [RCCSD(T)]. Their best estimated AEA is 24,212.75 cm^−1^. Recently, Mogren et al. [16] used Gaussian-3 theory to study the *R_e_* and *ω_e_* of the X^1^Σ^+^ state of SiN^−^. Furthermore, they derived the AEA (25,648.42 cm^−1^) and vertical electron affinity (VEA = 24,115.97 cm^−1^) at the MP2/6-31G* level. After summarizing these theoretical spectroscopic results [11,12,13,14,15,16], we firstly found that all of the calculations were mainly focused on the X^1^Σ^+^ and a^3^Σ^+^ states, and few results achieved high quality. Secondly, it was observed that no spin-orbit coupling (SOC) interactions were involved, though the SOC effect could influence the accurate prediction of spectroscopic properties [17,18]. Finally, it was found that no transition probabilities, such as Franck-Condon (FC) factors and the radiative lifetimes of the a^3^Σ^+^_1_ (*υ*′ = 0–2) to X^1^Σ^+^_0+_ transition, were calculated despite the fact that the transition probabilities were very useful in observing the corresponding states. Therefore, to improve the quality of the spectroscopic parameters of the anion, more accurate calculations should be done.

This paper is organized as follows. The methodology is briefly introduced in the next section. The PECs are reported in Section 3. The spectroscopic parameters and vibrational properties are predicted. The SOC effect on the spectroscopic parameters and vibrational levels is evaluated. The transition dipole moments (TDMs) between the a^3^Σ^+^_1_ and X^1^Σ^+^_0+_ states are determined. Calculations of FC factors and the radiative lifetimes of the a^3^Σ^+^_1_ to X^1^Σ^+^_0+_ transition are shown. Some conclusions are drawn in Section 4. The spectroscopic parameters, vibrational levels, and transition probabilities obtained here can be considered very reliable.

## 2. Methodology

The electron affinities of ground-state Si and N atoms are 11,207.24 and −560 cm^−1^ [19], respectively. Thus, the first dissociation channel of the SiN^−^ anion should be Si^−^(^4^S_u_) + N(^4^S_u_). The first and second excited states of Si^−^ anion are ^2^D_u_ and ^2^P_u_; their energy levels relative to the ground state (^4^S_u_) are approximately 6961.85 and (10,977.24 + *x^c^*)/2 cm^−1^ [19], respectively. The first excited state of the N atom is ^2^D_u_; its energy level relative to its ground state (^4^S_u_) is approximately 19,228.82 cm^−1^ [20]. Using the electron affinities and energy levels, we can determine that the second and third dissociation channels of the SiN^−^ anion are Si^−^(^2^D_u_) + N(^4^S_u_) and Si^−^(^2^P_u_) + N(^4^S_u_), respectively. With the help of group theory, 4 states (X^1^Σ^+^, a^3^Σ^+^, 1^5^Σ^+^, and 1^7^Σ^+^) were correlated to the first dissociation channel, 6 states (b^3^Δ, c^3^Π, d^3^Σ^+^, 2^5^Σ^+^, 1^5^Π, and 1^5^Δ) were correlated to the second dissociation limit, and 4 states (e^3^Σ^−^, 1^5^Σ^−^, f^3^Π, and 2^5^Π) were correlated to the third dissociation limit. These states and asymptotes are collected in Table 1. Using the approaches outlined above, we have determined the energy separation between each higher dissociation channel and the lowest one, i.e., Si^−^(^4^S_u_) + N(^4^S_u_). For reasons of comparison, we also collected the energy separation obtained using the icMRCI + Q/56 + CV + DK calculations and experiments [19] in Table 1. As seen in Table 1, the present results agree favorably with the measurements [19]. The results indicate that our calculations can properly describe the dissociation properties of SiN^−^.

The PECs were calculated using the complete active space self-consistent field (CASSCF) method followed by the internally contracted multireference configuration interaction (icMRCI) plus the Davidson modification (icMRCI + Q) approach [21,22] for internuclear separations from approximately 0.10 to 1.08 nm. As a result, the CASSCF was used as the reference wavefunction for the icMRCI calculations. In the calculations for the Si and N atoms, the basis sets aug-cc-pV5Z (AV5Z) and aug-cc-pV6Z (AV6Z) [23,24] were employed. The calculations were done with the MOLPRO 2010.1 program package [25] in the C_2*v*_ point group. The point spacing interval used for calculating the PECs is 0.02 nm for each state. To accurately determine each PEC, the point spacing interval used was further reduced to 0.002 nm for internuclear separations from approximately 0.11 to 0.50 nm; this is because the equilibrium separations fall into this range for all the bound and quasi-bound states involved. It should be noted that these point spacing intervals were suitable for all the calculations including the core–valence correlation, scalar relativistic corrections, and the SOC effect.

The molecular orbitals (MOs) used for the icMRCI calculations come from the CASSCF results. The state-averaged technique was used in the CASSCF calculations. To accurately determine the interaction between different PECs (such as avoided crossings), we put the 18 electronic states together into the calculations. Each state has the same weight factor of 0.0588235. In this paper, we only reported the PECs of 14 states arising from the first three dissociation limits. In the icMRCI calculations, we put the 8 outermost MOs (4a_1_, 2b_1_, and 2b_2_) into the active space. This corresponds to the 5-8σ, 2π, and 3π MOs in the anion. No additional MOs were included in the active space. That is, the 10 valence electrons were distributed into the 8 valence MOs of the SiN^−^ anion. Consequently, this active space was referred to as CAS (10, 8). The rest of the 12 inner electrons were put into the 6 lowest MOs (4a_1_, 1b_1_, and 1b_2_). For the AV6Z basis set, the total number of external orbitals is 368, corresponding to 126a_1_, 90b_1_, 90b_2_, and 62a_2_. In summary, we used 14 MOs (8a_1_, 3b_1_, and 3b_2_) to calculate the PECs of all the 14 Λ-S states.

Scalar relativistic correction was computed using the cc-pV5Z-DK basis set [26]. Its contribution to the total energy is denoted as DK. The core-valence correlation correction was calculated with the cc-pCVTZ basis set [27]. Its contribution to the total energy is denoted as CV. The SOC effect was determined by the state interaction method with the Breit-Pauli operator [28] at the level of the icMRCI theory with the all-electron cc-pCVTZ basis set. The all-electron cc-pCVTZ basis set with and without the Breit–Pauli operator was used to calculate the contribution to potential energy via the SOC effect. The difference between the two energies is the SOC splitting energy, which is denoted as SOC. The extrapolation of potential energy to the complete basis set (CBS) limit was performed with the AV5Z and AV6Z basis sets. The energy obtained from the extrapolation is denoted as 56. The extrapolation scheme [29] is as follows:

(1) $$\Delta E_{X}^{ref}=E_{\infty }^{ref}+A^{ref}X^{-\alpha }$$

(2) $$\Delta E_{X}^{corr}=E_{\infty }^{corr}+A^{corr}X^{-\beta }.$$

Here, $\Delta E_{X}^{ref}$ and $\Delta E_{X}^{corr}$ are the CASSCF and correlation energies obtained by the aug-cc-pVXZ basis set, respectively. $E_{\infty }^{ref}$ and $E_{\infty }^{corr}$ are the CASSCF and correlation energies extrapolated to the CBS limit, respectively. The extrapolation parameters *α* and *β* are taken as 3.4 and 2.4 for the Hartree–Fock and correlation energies [29], respectively.

With the PECs, the spectroscopic parameters *T_e_*, *R_e_*, *ω_e_*, *ω_e_x_e_*, *ω_e_у_e_*, *α_e_*, *B_e_*, and *D_e_* were evaluated. The meanings of these spectroscopic notations are explained in our earlier paper [30,31]. All the PECs were fitted to an analytical form by cubic splines. The rovibrational constants were first obtained from the analytic potential by solving the rovibrational Schrödinger equation, and the spectroscopic parameters were then evaluated by fitting the vibrational levels. 

## 3. Results and Discussion

**Figure 1:**
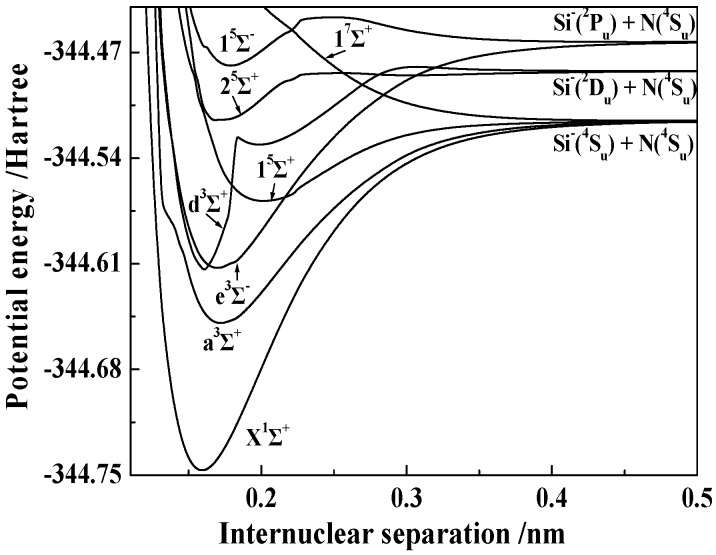
PECs of the 8 Λ-S states with the Σ symmetry of the SiN^−^ anion.

**Figure 2:**
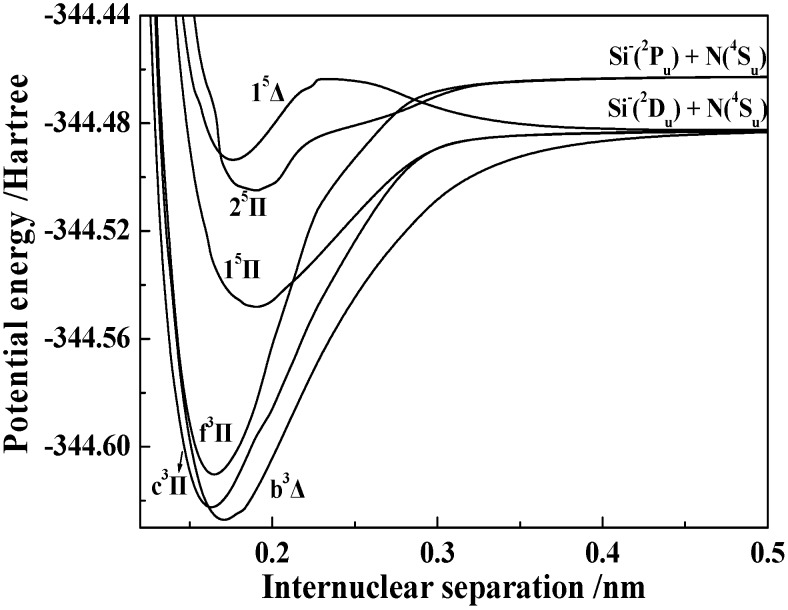
PECs of the 6 Λ-S states with the Π and Δ symmetries of the SiN^−^ anion.

Figure 1 and Figure 2 show the PECs of 14 electronic states obtained by the icMRCI + Q/56 + CV + DK calculations. To clearly display the details of each PEC, we plotted them only over a small internuclear separation range from approximately 0.11 to 0.50 nm. The dissociation channel of each state is also indicated in the figure.

From the PECs shown in Figure 1 and Figure 2, we can summarize the following features. (1) All the bound states (i.e., X^1^Σ^+^, a^3^Σ^+^, b^3^Δ, c^3^Π, e^3^Σ^−^, f^3^Π, 1^5^Σ^+^, 1^5^Π, and 2^5^Π) have a single well. (2) Two states, namely 1^5^Δ and 1^5^Σ^−^, each have a single well and one barrier. The potential energies of the two barriers are obviously larger than those at their respective dissociation limits. (3) Besides the 1^5^Δ and 1^5^Σ^−^ states, the d^3^Σ^+^ and 2^5^Σ^+^ states also have barriers on their PECs. In detail, the d^3^Σ^+^ state has a double well and two barriers; the 2^5^Σ^+^ state has a double well and one barrier, but the second well of the 2^5^Σ^+^ state is so shallow that it cannot be clearly distinguished. The barriers and the double wells of the d^3^Σ^+^ and 2^5^Σ^+^ states will be discussed in detail later in this paper. (4) The 1^7^Σ^+^ state is repulsive. (5) The avoided crossings are found in four paired states: the 2^3^Σ^+^ and 3^3^Σ^+^ states, the 2^5^Σ^+^ and 3^5^Σ^+^ states, the 1^5^Δ and 2^5^Δ states, and the 1^5^Σ^−^ and 2^5^Σ^−^ states. 

To better study the transition probabilities between two electronic states, we present the leading valence electronic configurations of all the bound and quasi-bound states near their respective internuclear equilibrium positions in Table S1 of the Supporting Information section. These were determined by the icMRCI/AV6Z calculations. Due to length limitations, we only tabulated these valence configurations in Table S1 if the coefficients-squared of the configuration-state function (CSF) were larger than 0.1.

### 3.1. Electron Affinity of SiN and the Stable States of SiN^−^

#### 3.1.1. Electron Affinity of SiN

At the icMRCI + Q/56 + CV + DK level of theory, the AEA and VEA of SiN are computed to be 23,262.27cm^−1^ and 22,766.75 cm^−1^, respectively. These correspond to the SiN (X^2^Σ^+^, *υ* = 0) + e^−^ → SiN^−^ (X^1^Σ^+^, *υ*′′ = 0) and SiN (X^2^Σ^+^, *υ* = 0) + e^−^ → SiN^−^ (X^1^Σ^+^, 0 < *υ*′′ < 1) transitions, respectively. Our AEA agrees well with the experimental result of 23,785.28 cm^−^^1^ [10]. Only the theoretical AEA obtained by Kerkines and Mavridis [15] is slightly closer to the experimental result than this one. In addition, the vertical detachment energy (VDE) of SiN^−^ was calculated at the icMRCI + Q/56 + CV + DK level of theory as 23,639.13cm^−^^1^. This corresponds to the SiN (X^2^Σ^+^, 0 < *υ*^+^ < 1) + e^−^ ← SiN^−^ (X^1^Σ^+^, *υ*′′ = 0) transition. The computed electron affinities adhere to the expected trend: VEA < AEA < VDE.

#### 3.1.2. Spectroscopic Parameters of the 13 Stable and Metastable Λ-S States of SiN^−^

Employing the PECs determined by the icMRCI + Q/56 + CV + DK calculations, we evaluated the spectroscopic parameters of 13 Λ-S states. For the purposes of this discussion and due to length limitations, the spectroscopic parameters of the 13 Λ-S states are given in Table S2, along with the experimental only [10] and other theoretical [11,12,13,14,15,16] spectroscopic results.

The ground state X^1^Σ^+^ of the SiN^−^ anion is mainly characterized by the closed-shell electronic configuration 5σ^2^6σ^2^7σ^2^2π^4^3π^0^8σ^0^ (0.827). A group of experimental work [10] and six groups of calculations [11,12,13,14,15,16] reported the spectroscopic parameters of this state. Values of *R_e_* and *ω_e_* for this state obtained in this work deviate from the experimental values [10] by only 0.00117 nm and 8.87 cm^−1^, respectively. Using the values of *ω_e_*, *ω_e_x_e_* and *ω_e_y_e_* obtained here in combination with the equation *D_e_* = *D*_0_ + *ω_e_*/2 − *ω_e_x_e_*/4 + *ω_e_y_e_*/8, we determined the ground state *D*_0_ to be approximately 6.2466 eV. Obviously, the present *D*_0_ is close to the experimental values [10] as the deviation is only 0.0066 eV. We found, as shown in Table 3, that only the theoretical *R_e_* values obtained by Peterson and Woods [11] and Kalcher [13] are slightly closer to the measurements [10] than what we have obtained. This state has a well depth of approximately 50,592.79 cm^−1^. It has 81 vibrational states, as tabulated in Table S3. Nevertheless, X^1^Σ^+^ (*υ*′′ = 24) lies 23,613.44cm^−1^ above the X^1^Σ^+^ (*υ*′′ = 0), which is larger than the calculated AEA. This means the X^1^Σ^+^ *υ*′′ ≥ 24 vibrational levels are difficult to observe in a spectroscopy experiment.

The a^3^Σ^+^, b^3^Δ, c^3^Π, e^3^Σ^−^, f^3^Π, 1^5^Σ^+^, 1^5^Π, and 2^5^Π states also possess the single reference character near the equilibrium position. The dominant electronic configurations of the a^3^Σ^+^, b^3^Δ, e^3^Σ^−^, and 1^5^Σ^+^ states arise from the 2π → 3π singlet electronic excitation of the ground state. The dominant electronic configurations of the c^3^Π and f^3^Π states are generated predominantly from the 7σ → 3π and 2π → 8σ singlet electronic excitations of the ground state, respectively, whereas the dominant electronic configurations of the 1^5^Π and 2^5^Π states come from the 2π → 3π, 7σ → 3π, and 2π → 3π, 2π → 8σ double electronic excitations of the ground state, respectively. 

Two groups of calculations have been performed [13,15], but no measurements on the spectroscopic parameters of the a^3^Σ^+^ state had been reported. Compared with the results obtained by Kalcher [13], the present *R*_e_ and *D_e_* results are slightly smaller, but the present *ω_e_* and *T_e_* values are obviously larger. In addition, the present *T*_e_ value is obviously smaller, but the present *R_e_* and *D_e_* values are larger than the previous results [15]. It should be noted that the previous results [13,15] were calculated using a small basis set [13] and the single reference method [15], whereas the present ones are derived by the icMRCI + Q method with extrapolation to the CBS limit and including various corrections. For this reason, we believe the present results should be more accurate and reliable [13,15]. To date, no experimental or other theoretical spectroscopic parameters have been reported in the literature for the b^3^Δ, c^3^Π, e^3^Σ^−^, f^3^Π, 1^5^Σ^+^, 1^5^Π, or 2^5^Π states.

For the a^3^Σ^+^ state, the depth of the well is approximately 29,492.12 cm^−1^. It has 68 vibrational states, as tabulated in Table S3. However, the a^3^Σ^+^ (*υ*′ = 3) lies 23,604.85 cm^−1^ above the X^1^Σ^+^ (*υ*′′ = 0), and is also larger than the calculated AEA. Hence, the a^3^Σ^+^ (*υ*′ ≥ 3) would be metastable towards autodetachment.

As with X^1^Σ^+^ (*υ*′′ ≥ 24) and a^3^Σ^+^ (*υ*′ ≥ 3), the b^3^Δ, c^3^Π, e^3^Σ^−^, f^3^Π, 1^5^Σ^+^, 1^5^Π, and 2^5^Π states are metastable states. 

Two-pair avoided crossings are located at approximately *R* = 0.2284 nm (between the 1^5^Δ and 2^5^Δ states) as well as at approximately *R* = 0.2604 nm (between the 1^5^Σ^−^ and 2^5^Σ^−^ states). Therefore, the 1^5^Δ and 1^5^Σ^−^ states have a potential well induced by the avoided crossings in the range of *R <* 0.2284 and *R <* 0.2604 nm, respectively. They are repulsive at larger internuclear distances. The potential energy at the top of the barrier of each state is higher than that at their respective dissociation limits by approximately 4250.02 and 3750.36 cm^−1^, respectively. Thus, both the dissociation energy and well depth of each state are relative to their respective barriers and are equal to 6696.83 and 7213.21 cm^−1^, respectively. The 1^5^Δ and 1^5^Σ^−^ states possess 11 and 12 vibrational states, respectively. These vibrational levels are summarized in Table S4. The dominant electronic transitions between the 1^5^Δ and 1^5^Π states and between the 1^5^Σ^−^ and 1^5^Π states can be regarded as 3π to 8σ promotions. 

The d^3^Σ^+^ state has a double well and two barriers. The two barriers lie at approximately 0.1824 and 0.3004 nm, respectively. They are formed by the avoided crossings of the d^3^Σ^+^ and 3^3^Σ^+^ states. The potential energy at the top of the first barrier is lower, whereas that of the second barrier is higher than that at the dissociation limit for the d^3^Σ^+^ state. Therefore, the depths of the two wells should be relative to the first barrier, but their dissociation energies must be relative to the second barrier. The depth of the first well is approximately 19,808.90 cm^−1^. It has 12 vibrational states, as listed in Table S4. The depth of the second well is approximately 1355.04 cm^−1^. It has three vibrational states, as presented in Table S4.

The 2^5^Σ^+^ state has a double well and one barrier. The barrier is generated by the avoided crossing of this state with the 3^5^Σ^+^ state at approximately 0.2604 nm. For the 2^5^Σ^+^ state, the potential energy at the top of the barrier is lower than that at the dissociation limit. Thus, the depths of the double well should be relative to the barrier, whereas the dissociation energies of the double well must be relative to the dissociation limit. For the double well of the 2^5^Σ^+^ state, the well depths are 6970.08 and 450.14 cm^−1^, with 11 and 5 vibrational levels, respectively. These vibrational levels are presented in Table S4. 

As with the b^3^Δ, c^3^Π, e^3^Σ^−^, f^3^Π, 1^5^Σ^+^, 1^5^Π, and 2^5^Π states, neither theoretical nor experimental studies have been reported in the literature regarding the 1^5^Δ, 1^5^Σ^−^, d^3^Σ^+^, and 2^5^Σ^+^ metastable states. 

### 3.2. Spectroscopic Parameters and Vibrational Levels of the 45 Ω Bound States

When the SOC is taken into account, the Si^−^(^4^S_u_) + N(^4^S_u_) dissociation channel splits into one dissociation asymptote. Each of the Si^−^(^2^D_u_) + N(^4^S_u_) and Si^−^(^2^P_u_) + N(^4^S_u_) dissociation limits splits into two asymptotes. These Ω states and their dissociation asymptotes are listed in Table 2. In employing the icMRCI + Q/56 + CV + DK + SOC calculations, we determined the energy separations between each higher asymptote and the lowest one, i.e., Si^−^(^4^S_u_) + N(^4^S_u_). The dissociation relationships for the possible Ω states and corresponding energy separations are also listed in Table 2. Simultaneously, we also collected the corresponding experimental energy separations [19] for comparison. As seen in Table 2, the energy separations obtained in this paper agree favorably with the experimental values [19].

The 14 Λ-S states are split into 49 Ω states. In detail, 10 Ω states (X^1^Σ^+^_0+_, a^3^Σ^+^_1_, a^3^Σ^+^_0__−_, 1^5^Σ^+^_2_, 1^5^Σ^+^_1_, 1^5^Σ^+^_0+_, 1^7^Σ^+^_0__−_, 1^7^Σ^+^_1_, 1^7^Σ^+^_2_, and 1^7^Σ^+^_3_) arise from the Si^−^(^4^S_3/2_) + N(^4^S_3/2_) channel, 10 Ω states (c^3^Π_0__−_, c^3^Π_0+_, c^3^Π_1_, c^3^Π_2_, 1^5^Π_3_, 1^5^Π_2_, 1^5^Π_1_, 1^5^Π_0+_, 1^5^Π_0__−_, and 1^5^Π_−1_) are produced from the Si^−^(^2^D_3/2_) + N(^4^S_3/2_) channel, 14 Ω states (b^3^Δ_1_, b^3^Δ_2_, b^3^Δ_3_, d^3^Σ^+^_0__−_, d^3^Σ^+^_1_, 1^5^Δ_0__−_, 1^5^Δ_0+_, 1^5^Δ_1_, 1^5^Δ_2_, 1^5^Δ_3_, 1^5^Δ_4_, 2^5^Σ^+^_2_, 2^5^Σ^+^_1_, and 2^5^Σ^+^_0+_) are associated with the Si^−^(^2^D_5/2_) + N(^4^S_3/2_) asymptote, 5 Ω states (2^5^Π_−1_, 2^5^Π_0__−_, 2^5^Π_0+_, 2^5^Π_1_, and 2^5^Π_2_) are yielded from the Si^−^(^2^P_1/2_) + N(^4^S_3/2_) asymptote, and 10 Ω states (e^3^Σ^−^_0+_, e^3^Σ^−^_1_, f^3^Π_2_, f^2^Π_1_, f^3^Π_0+_, f^3^Π_0__−_, 2^5^Π_3_, 1^5^Σ^−^_0__−_, 1^5^Σ^−^_1_, and 1^5^Σ^−^_2_) belong to the Si^−^(^2^P_3/2_) + N(^4^S_3/2_) channel. Of these 49 Ω states, only the 1^7^Σ^+^_0__−_, 1^7^Σ^+^_1_, 1^7^Σ^+^_2_, and 1^7^Σ^+^_3_ states are repulsive.

To conveniently discuss the spectroscopic parameters and vibrational levels of these Ω states, we divide them into three types according to their symmetries. The first group comprises 16 Ω states, which arise from the X^1^Σ^+^, a^3^Σ^+^, d^3^Σ^+^, e^3^Σ^−^, 1^5^Σ^+^, 2^5^Σ^+^, and 1^5^Σ^−^ states. The second group comprises 20 Ω states, which come from the c^3^Π, f^3^Π, 1^5^Π, and 2^5^Π states. The third group comprises nine Ω states, which are generated from the b^3^Δ and 1^5^Δ states. 

#### 3.2.1. Spectroscopic and Vibrational Properties of 16 Ω States with the Σ Symmetry

Using the PECs obtained by the icMRCI + Q/56 + CV + DK + SOC calculations, we evaluated the *T_e_*, *D_e_*, *R_e_*, and *ω_e_* values of these 16 Ω states with Σ symmetry. The spectroscopic parameters are presented in Table S5. For reasons of discussion, the leading Λ-S state compositions of each Ω state near their respective equilibrium positions are also presented in Table S5. For clarity, we neglected the Λ-S states that contribute less than 0.08% to the total Λ-S state composition.

The X^1^Σ^+^ state does not split when the SOC effect is included. The X^1^Σ^+^_0+_ state completely consists of the X^1^Σ^+^ state in the FC region. Comparing *R_e_*, *ω_e_*, and *D_e_* values collected in Tables S2 and S5, we confirmed that the SOC effect on these spectroscopic parameters can be negligible. Additionally, the vibrational levels of X^1^Σ^+^_0+_ states are almost equal to the corresponding values of the X^1^Σ^+^ state.

The SOC interaction causes the a^3^Σ^+^ and e^3^Σ^−^ states to split into four Ω states: a^3^Σ^+^_1_, a^3^Σ^+^_0__−_, e^3^Σ^−^_0+_, and e^3^Σ^−^_1_, in the order of increasing energy. The a^3^Σ^+^ state is inverted, whereas the e^3^Σ^−^_1_ state is regular when the SOC effect is taken into account. The SOC effect on the *R_e_*, *ω_e_*, and *D_e_* values of these two states is small. This is consistent with the fact that the Λ-S state compositions of each Ω state are almost pure near their respective equilibrium positions. The SOC splitting energies of a^3^Σ^+^ and e^3^Σ^−^ states are only 0.87 and 0.88 cm^−1^, respectively. This is not large. In comparing the *T_e_*, *R_e_*, *ω_e_*, and *D_e_* values of each Ω state with those seen in Table S2, we affirm that the difference between them is small. This shows that the SOC effect is very small in the spectroscopic parameters of these Ω states. In addition, the SOC effect on their vibrational levels is also small. 

Under the SOC effect, the d^3^Σ^+^ state is regular and still has a double well. As seen in Table S5, for the double well, the Λ-S state compositions of each Ω state are pure around their respective equilibrium positions. Accordingly, the spectroscopic parameters and vibrational levels of the d^3^Σ^+^_0__−_ and d^3^Σ^+^_1_ states are almost equal to those of the d^3^Σ^+^ state for both the first and second well. 

Each of the 1^5^Σ^+^ and 1^5^Σ^−^ states splits into three Ω states with the SOC effect taken into account. As seen in Table S5, the Λ-S state compositions of each Ω state are almost pure around their respective equilibrium positions. Accordingly, the SOC effect on their spectroscopic parameters is small. Through comparison, we confirm that the SOC effect on their vibrational levels is also very small. In addition, the 1^5^Σ^+^ state is inverted when the SOC effect is included. 

The 2^5^Σ^+^_2_, 2^5^Σ^+^_1_, and 2^5^Σ^+^_0+_ states have a double well. The *T_e_* of the 2^5^Σ^+^_2_ state is smaller than that of the 2^5^Σ^+^_0+_ state for both the first and second well. Therefore, the 2^5^Σ^+^ state is inverted when the SOC effect is taken into account. For the first well, as seen in Table S5, the Λ-S state compositions of each Ω state are pure near the equilibrium positions. This is also true with the 1^5^Σ^+^ and 1^5^Σ^−^ states. The SOC effect on the spectroscopic parameters and vibrational levels of all the Ω states is tiny. For the second well, the Λ-S state compositions of the three Ω states slightly mix with the c^3^Π and 1^5^Π states near their respective equilibrium positions. The largest deviations of *R_e_*, *ω_e_*, and *D_e_* values of each Ω state from those originating from the 2^5^Σ^+^ state are 0.00019 nm, 1.275 cm^−1^, and 0.0003 eV, respectively. The SOC splitting energies between the two neighboring Ω states from 2^5^Σ^+^_2_ to 2^5^Σ^+^_0+_ are only 0.65 and 0.22 cm^−1^, respectively. Each Ω state has five vibrational levels, which are almost equal to those of the second well of the 2^5^Σ^+^ state. 

In conclusion, (1) the a^3^Σ^+^, 1^5^Σ^+^ and 2^5^Σ^+^ states are inverted with the SOC effect taken into account. (2) The SOC effect to the spectroscopic parameters and the vibrational levels of all the Σ states is small.

#### 3.2.2. Spectroscopic and Vibrational Properties of 20 Ω States with the Π Symmetry

Using the PECs obtained by the icMRCI + Q/56 + CV + DK + SOC calculations, we evaluated *T_e_*, *R_e_*, *ω_e_*, and *D_e_* values of the 20 Ω states with the Π symmetry. The spectroscopic parameters are tabulated in Table S6. As with Table S5, also presented the leading Λ-S state compositions of each Ω state around their respective equilibrium positions.

The c^3^Π state splits into four Ω states (i.e., c^3^Π_0−_, c^3^Π_0+_, c^3^Π_1_, and c^3^Π_2_) when the SOC effect is accounted for. The SOC splitting energies between two consecutive Ω states from the c^3^Π_0−_ to c^3^Π_2_ states are 0.44, 47.19, and 47.62 cm^−1^, respectively. These are relatively large when compared with those of other states. The SOC effect on *R_e_*, *ω_e_* and *D_e_* values of each Ω state is not large. The largest deviations of *R_e_*, *ω_e_*, and *D_e_* values of all four Ω states of the split c^3^Π state are 0.00002 nm, 0.274 cm^−1^, and 0.0059eV, respectively. In comparison, we can confirm that the SOC effect on the vibrational levels of these Ω states is also small.

Under the SOC effect, the f^3^Π also splits into four Ω states (f^3^Π_0−_, f^3^Π_0+_, f^3^Π_1_, and f^3^Π_2_). Their energies increase in the order of f^3^Π_0−_, f^3^Π_0+_, f^3^Π_1_, and f^3^Π_2_ in the range of *R <* 0.1644 nm. However, the order of f^3^Π_0−_, f^3^Π_0+_, f^3^Π_1_, and f^3^Π_2_ changes in the internuclear distance region from 0.1644 to 1.08 nm. This phenomenon leads to the obvious change of the PECs for the f^3^Π_0__−_, f^3^Π_0+_, and f^3^Π_2_ states. As a result, the spectroscopic parameters and the vibrational levels of f^3^Π_0__−_, f^3^Π_0+_, and f^3^Π_2_ states are obviously different from those of the corresponding Λ-S state.

Each of the 1^5^Π and 2^5^Π states splits into six Ω states with the SOC effect taken into account, and the 1^5^Π state is inverted. For the 1^5^Π state, the largest deviations of *R_e_*, *ω_e_*, and *D_e_* of each Ω state from those of the 1^5^Π state are 0.00001 nm, 0.298 cm^−1^, and 0.0047eV, respectively; the SOC splitting energies between the two neighboring Ω states from the 1^5^Π_3_ to the 1^5^Π_−1_ are only 17.99, 18.22, 18.31, 0.13, and 18.65 cm^−1^, respectively; on the whole, the SOC effect on the splitting energies is not large; each Ω state has 38 vibrational levels, which are almost equal to those of the first well of 1^5^Π state. For the 2^5^Π state, the Λ-S state compositions of each Ω state are pure near the equilibrium positions; as with the c^3^Π and 1^5^Π states, the SOC effect on *R_e_*, *ω_e_* and *D_e_* of all the Ω states is tiny; each Ω state has the 36 vibrational states, in which vibrational levels are almost equal to those of the 2^5^Π state; the energy splitting of 2^5^Π_−1_ to 2^5^Π_0__−_, 2^5^Π_0__−_ to 2^5^Π_0+_, 2^5^Π_0+_ to 2^5^Π_1_, 2^5^Π_1_ to 2^5^Π_2_, and 2^5^Π_2_ to 2^5^Π_3_ are 22.17, 0.22, 21.95, 22.16, and 22.17cm^−1^, respectively, which are not large; thus, we affirm that the SOC effect on the spectroscopic and vibrational properties of all the 6 Ω states is very small. 

Our conclusions are as follows: (1) the SOC effect on the spectroscopic parameters is tiny except for the f^3^Π state and the spitting energies of c^3^Π state; (2) the 1^5^Π state is inverted, and the f^3^Π state is also inverted in the internuclear distance range from 0.1644 to 1.08 nm with the SOC effect taken into account; (3) the SOC effect on the vibrational levels of all the Π states is small expect for the f^3^Π_0__−_, f^3^Π_0+_, and f^3^Π_2_ states.

#### 3.2.3. Spectroscopic and Vibrational Properties of Nine Ω States with the Δ Symmetry

Using the PECs obtained by the icMRCI + Q/56 + CV + DK + SOC calculations, we evaluated the *T_e_*, *D_e_*, *R_e_*, and *ω_e_* values of nine Ω states with the Δ symmetry. The spectroscopic parameters are presented in Table S7. As with Tables S5 and S6, the leading Λ-S state compositions of each Ω state around their respective internuclear equilibrium positions are also tabulated in Table S7.

The b^3^Δ state splits into three Ω states when the SOC effect is taken into account. For the three Ω states, the energy arrangement from low to high is b^3^Δ_3_, b^3^Δ_2_, and b^3^Δ_1_. The *T_e_*, *R_e_*, *ω_e_*, and *D_e_* values of each Ω state are very close to those of the b^3^Δ state. Each Ω state has 70 vibrational states, the vibrational levels of which are almost equal to those of the b^3^Δ state. As a result, we conclude that the SOC effect on the spectroscopic parameters and vibrational states is tiny. 

The 1^5^Δ state splits into the 1^5^Δ_0__−_, 1^5^Δ_0+_, 1^5^Δ_1_, 1^5^Δ_2_, 1^5^Δ_3_, and 1^5^Δ_4_ states when the SOC effect is included, as tabulated in Table S7. The Λ-S state compositions of each Ω state are almost pure around the internuclear equilibrium positions. Consequently, the SOC effect on the spectroscopic parameters and vibrational levels of each Ω state are inconspicuous. 

In conclusion, (1) the SOC effect on the spectroscopic parameters and vibrational properties of the b^3^Δ and 1^5^Δ states is very small, and (2) the b^3^Δ state is inverted.

### 3.3. Transition Properties

**Figure 3:**
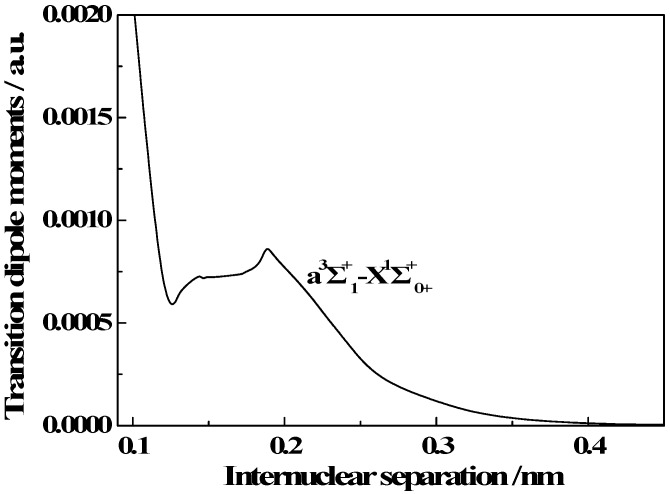
TDMs versus internuclear separations of transitions from the a^3^Σ^+^_1_ state to the X^1^Σ^+^_0+_ state.

The TDMs between the a^3^Σ^+^_1_ and X^1^Σ^+^_0+_ states were obtained with the Breit-Pauli Hamiltonian in combination with the all-electron CVTZ basis set at the level of the icMRCI theory. The curves of TDM versus internuclear separation are depicted in Figure 3. As with the PECs, to clearly show the main features of each TDM curve, we display them over a small range of internuclear separations. The figure clearly shows that the TDMs of the transitions from the a^3^Σ^+^_1_ state to the X^1^Σ^+^_0+_ state very small in the FC region. This is consistent with the fact that the triplet-singlet transitions are forbidden. In addition, the TDMs go to the zero asymptote when the internuclear distance is larger than 0.4 nm.

Using the PECs and TDMs obtained here, we calculated the FC factors and Einstein coefficients of the a^3^Σ^+^_1_ (*υ*′ = 0–2) to X^1^Σ^+^_0+_ with the LEVEL program [32]. We only list the lowest 10 vibrational levels of the X^1^Σ^+^_0+_ state due to the length limitation. For the purposes of this discussion, we present these results in Table 3. Table 4 lists the radiative lifetimes for the a^3^Σ^+^_1_ (*υ*′ = 0–2) to X^1^Σ^+^_0+_ transition. Overall, the a^3^Σ^+^_1_ states are not easy to detect spectroscopically by observing these transitions. This can be explained based on the data in Table 3 and Table 4. As presented in Table 3 and Table 4, almost all of the Einstein coefficients of the a^3^Σ^+^_1_ to X^1^Σ^+^_0+_ transition are very small and their radiative lifetimes are very long. That is, these transitions are weak. 

## 4. Conclusions

In this work, we calculated the PECs of 14 Λ-S states of the SiN^−^ anion using the icMRCI + Q/56 + CV + DK approach, computed the TDMs of the a^3^Σ^+^_1_ to X^1^Σ^+^_0+_ transition using the icMRCI approach with the all-electron CVTZ basis set, and determined the spectroscopic parameters of 49 Ω states employing the icMRCI + Q/56 + CV + DK + SOC method. The spectroscopic parameters and vibrational levels were evaluated. In addition, the transition probabilities of a^3^Σ^+^_1_ to X^1^Σ^+^_0+_ were studied. These results were compared in detail with those reported in the literature. Excellent agreement is found between these results and those of other measurements. Of these 14 states, only the 1^7^Σ^+^ state is repulsive. The avoided crossings exist between the d^3^Σ^+^ and 3^3^Σ^+^ states, between the 2^5^Σ^+^ and 3^5^Σ^+^ states, between the 1^5^Δ and 2^5^Δ states, and between the 1^5^Σ^−^ and 2^5^Σ^−^ states. The X^1^Σ^+^ (*υ*′′ ≥ 24), a^3^Σ^+^ (*υ*′ ≥ 3), b^3^Δ, c^3^Π, d^3^Σ^+^, e^3^Σ^−^, f^3^Π, 1^5^Σ^+^, 1^5^Π, 2^5^Σ^+^, 2^5^Π, 1^5^Δ, and 1^5^Σ^−^ states are metastable states. The d^3^Σ^+^, b^3^Δ, f^3^Π, 1^5^Σ^+^, 1^5^Π, and 2^5^Σ^−^ states are inverted when the SOC effect is included. The SOC effect on the spectroscopic parameters and vibrational levels is small except for in the f^3^Π state and the spitting energies of c^3^Π state. The spectroscopic parameters, vibrational levels, and transition probabilities obtained in this paper can be considered very reliable and can be employed as helpful guidelines for detecting these states in an appropriate spectroscopy experiment in the near future. In addition, the data provided in this work should assist in spectral searches for this anion in the interstellar medium.

## Figures and Tables

**Table 1:** Dissociation relationships of the 14 states generated from the first three dissociation channels of the SiN^−^ anion.

Dissociation Channel	Electronic State	Relative Energy/cm^−1^	
		This Work *^a^*	Exp. [19]
Si^−^ (^4^S_u_) + N(^4^S_u_)	X^1^Σ^+^, a^3^Σ^+^, 1^5^Σ^+^, 1^7^Σ^+^	0.0	0.0
Si^−^ (^2^D_u_) + N(^4^S_u_)	b^3^Δ, c^3^Π, d^3^Σ^+^, 2^5^Σ^+^, 1^5^Π, 1^5^Δ	6973.48	6961.85 *^b^*
Si^−^ (^2^P_u_) + N(^4^S_u_)	e^3^Σ^−^, 1^5^Σ^−^, f^3^Π, 2^5^Π	10,974.90	(10,977.24 + *x ^c^*)/2

*^a^* Obtained using the icMRCI + Q/56 + CV + DK calculations; *^b^* obtained by averaging the atomic energy levels of the ^2^D_3/2_ and ^2^D_5/2_ states; *^c^* no experimental energy level of ^2^P_3/2_ state obtained by Andersen et al. [19].

**Table 2:** Dissociation relationships of possible Ω states yielded from the first three dissociation channels of the SiN^−^ anion.

Atomic State	Possible Ω States	Relative Energy/cm^−1^	
		This Work *^a^*	Exp. [19]
Si^−^ (^4^S_3/2_) + N(^4^S_3/2_)	0^−^ (2), 0^+^(2), 1(3), 2(2), 3	0.00	0.00
Si^−^ (^2^D_3/2_) + N(^4^S_3/2_)	−1, 0^−^(2), 0^+^(2), 1(2), 2(2), 3	6968.32	6954.81
Si^−^ (^2^D_5/2_) + N(^4^S_3/2_)	0^−^(2), 0^+^(2), 1(4), 2(3), 3(2), 4	6978.63	6968.89
Si^−^ (^2^P_1/2_) + N(^4^S_3/2_)	−1, 0^−^, 0^+^, 1, 2	10,970.08	10,977.24
Si^−^ (^2^P_3/2_) + N(^4^S_3/2_)	0^−^(2), 0^+^(2), 1(3), 2(2), 3	10,979.76	*x ^b^*

*^a^* Obtained by the icMRCI + Q/56 + CV + DK + SOC calculations; *^b^* no experimental energy level of ^2^P_3/2_ state obtained by Andersen et al. [19].

**Table 3:** FC factors (1st line) and Einstein coefficients (s^−1^, 2nd line) for the a^3^Σ^+^_1_ to X^1^Σ^+^_0+_ transition.

State to State	*υ*′′ = 0	*υ*′′ = 1	*υ*′′ = 2	*υ*′′ = 3	*υ*′′ = 4	*υ*′′ = 5	*υ*′′ = 6	*υ*′′ = 7	*υ*′′ = 8	*υ*′′ = 9
a^3^Σ^+^_1_ to X^1^Σ^+^_0+_										
*υ*′ = 0	0.0970	0.2393	0.2796	0.2068	0.1099	0.0457	0.0159	0.0047	0.0011	0.0002
	0.3370	0.7136	0.7129	0.4506	0.2054	0.0736	0.0220	0.0056	0.0012	0.0002
*υ*′ = 1	0.1886	0.1666	0.0131	0.0455	0.1663	0.1929	0.1328	0.0644	0.0230	0.0057
	0.7245	0.5493	0.0359	0.1140	0.3519	0.3484	0.2064	0.0870	0.0274	0.0062
*υ*′ = 2	0.2373	0.0262	0.0709	0.1362	0.0207	0.0289	0.1378	0.1661	0.1101	0.0479
	1.0072	0.0954	0.2258	0.3692	0.0462	0.0613	0.2448	0.2544	0.1468	0.0556

**Table 4:** Radiative lifetime values of the transition from the a^3^Σ^+^_1_ (*υ*′= 0–2) excited Ω states to the X^1^Σ^+^_0+_ state for the SiN^−^ anion.

	Radiative Lifetimes		
Transitions	*υ*′ = 0	*υ*′ = 1	*υ*′ = 2
a^3^Σ^+^_1_ to X^1^Σ^+^_0+_ (ms)	396.5	407.9	396.4

## Supplementary Materials

The supplementary materials are available online.
