# Supplementary material for: icMRCI+Q Study of the Spectroscopic Properties of the 14 Λ-S and 49 Ω States of the SiN− Anion in the Gas Phase
Source: Molecules. 2018 Jan 20;23(1):210. doi: 10.3390/molecules23010210 (PMC6017747; doi:10.3390/molecules23010210)
Supplement: Supplementary file 1 [file molecules-23-00210-s001.pdf]

molecules

MDPI

Supplementary Information

icMRCI+Q study of the Spectroscopic Properties of the 14 Λ-S and 49 Ω States of the SiN⁻ Anion in the Gas Phase

Wei Xing[1,2], Jin-feng Sun[1,3*], De-heng Shi[3], and Zun-lue Zhu[3]

[1]School of Materials Science and Engineering, Henan University of Science and Technology, Luoyang 471023, China
[2]College of Physics and Electronic Engineering, Xinyang Normal University, Xinyang 464000, China
[3]College of Physics and Material Science, Henan Normal University, Xinxiang 453007, China

Table S1. Leading valence configurations of the 13 states near the equilibrium positions.

State	Dominant valence configuration	State	Dominant valence configuration
$X^1\Sigma^+$	$5\sigma^2 6\sigma^2 7\sigma^2 2\pi^4 3\pi^0 8\sigma^0$ (0.827)[a].	$1^5\Sigma^-$	$5\sigma^2 6\sigma^2 7\sigma^1 2\pi^3 3\pi^1 8\sigma^1$ (0.853).
$a^3\Sigma^+$	$5\sigma^2 6\sigma^2 7\sigma^2 2\pi^3 3\pi^1 8\sigma^0$ (0.839).	$2^5\Sigma^+$	
$b^3\Delta$	$5\sigma^2 6\sigma^2 7\sigma^2 2\pi^3 3\pi^1 8\sigma^0$ (0.846).	1st well	$5\sigma^2 6\sigma^2 7\sigma^1 2\pi^3 3\pi^1 8\sigma^1$ (0.827).
$e^3\Sigma^-$	$5\sigma^2 6\sigma^2 7\sigma^2 2\pi^3 3\pi^1 8\sigma^0$ (0.826).	2nd well	$5\sigma^2 6\sigma^2 7\sigma^1 2\pi^3 3\pi^1 8\sigma^1$ (0.354);
$c^3\Pi$	$5\sigma^2 6\sigma^2 7\sigma^1 2\pi^4 3\pi^1 8\sigma^0$ (0.817).		$5\sigma^2 6\sigma^2 7\sigma^1 2\pi^2 3\pi^2 8\sigma^1$ (0.203);
$f^3\Pi$	$5\sigma^2 6\sigma^2 7\sigma^2 2\pi^3 3\pi^0 8\sigma^1$ (0.829).		$5\sigma^2 6\sigma^2 7\sigma^1 2\pi^1 3\pi^3 8\sigma^1$ (0.161);
$1^5\Sigma^+$	$5\sigma^2 6\sigma^2 7\sigma^2 2\pi^2 3\pi^2 8\sigma^0$ (0.871).		$5\sigma^2 6\sigma^2 7\sigma^2 2\pi^2 3\pi^2 8\sigma^0$ (0.154).
$1^5\Pi$	$5\sigma^2 6\sigma^2 7\sigma^1 2\pi^3 3\pi^2 8\sigma^0$ (0.834).	$d^3\Sigma^+$	
$2^5\Pi$	$5\sigma^2 6\sigma^2 7\sigma^2 2\pi^2 3\pi^1 8\sigma^1$ (0.850).	1st well	$5\sigma^2 6\sigma^2 7\sigma^1 2\pi^4 3\pi^0 8\sigma^1$ (0.794).
$1^5\Delta$	$5\sigma^2 6\sigma^2 7\sigma^1 2\pi^3 3\pi^1 8\sigma^1$ (0.821).	2nd well	$5\sigma^2 6\sigma^2 7\sigma^2 2\pi^2 3\pi^2 8\sigma^0$ (0.847).

[a]Values in parentheses are the coefficients squared of CSF associated with the electronic configuration.

Table S2. Spectroscopic parameters obtained by the icMRCI + Q/ 56 + CV + DK calculations for the 13 states.

	T_e/cm⁻¹	R_e/nm	ω_e/cm⁻¹	$\omega_e x_e$/cm⁻¹	$10^3\omega_e y_e$/cm⁻¹	B_e/cm⁻¹	$10^3\alpha_e$/cm⁻¹	D_e/eV
$X^1\Sigma^+$	0.0	0.15923	1138.87	5.999	8.710	0.7124	5.227	6.3170
Exp. [10]	0.0	0.1604(5)	1130(20)	---	---	---	---	6.24(0.14)[a]
Cal. [11][b]	0.0	0.16024	1106.3	8.29	---	---	6.038	6.3659[c]
Cal. [12][d]	0.0	0.1586	1171.2[e]					
Cal. [13]	0.0	0.16045	1120.0	6.01	---	0.702		
Cal. [14][f]	0.0	0.1589	1183	7.16	-9.1	0.712	5.58	6.06
Cal.[15][g]	0.0	0.15908	---	---	---	---	---	6.2748
Cal. [16]	0.0	0.1790	1322					
$a^3\Sigma^+$	21412.16	0.17200	828.713	51.45	13315	0.6126	18.27	3.6841
Cal. [13]	20333.23	0.17342	820.5	5.86	---	0.601		
Cal. [15][g]	22319.1	0.17116	---	---	---	---	---	3.5082
$b^3\Delta$	26279.67	0.17073	854.332	61.70	13484	0.6251	23.99	3.9603
$c^3\Pi$	27300.89	0.16308	969.192	6.513	505.6	0.6792	6.293	3.8270
$d^3\Sigma^+$								
1st well	28926.10	0.15982	2177.06	227.8	25918	0.7101	12.21	3.7100
2nd well	47379.96	0.19867	537.125	3.290	90.53	0.4578	4.774	1.4220
$e^3\Sigma^-$	29433.30	0.16968	861.174	53.81	8993	0.6335	26.16	4.1084

$f^3\Pi$	29974.31	0.16475	1001.15	9.485	211.8	0.6658	7.289	4.0374
$1^5\Sigma^+$	39168.98	0.20130	483.760	6.480	3275	0.4457	8.253	1.4876
$1^5\Pi$	43629.80	0.19042	615.771	14.62	2194	0.4998	3.611	1.7937
$2^5\Sigma^+$								
1st well	50898.14	0.16780	753.818	121.9	38346	0.6091	13.91	0.8841
2nd well	57417.42	0.30045	124.233	4.794	502.7	0.2002	5.443	0.0757
$2^5\Pi$	53009.27	0.18879	766.246	126.9	25373	0.5102	4.976	1.1810
$1^5\Delta$	55587.44	0.17620	738.400	6.376	90.89	0.5818	6.443	0.8303
$1^5\Sigma^-$	58859.80	0.17882	724.024	6.678	895.1	0.5651	8.296	0.8943

Note: [a]D_0; [b]MP4SDQ/66 CGTOs calculations; [c]MP4SDTQ/93 CGTOs calculations; [d]SDCI+Q calculations; [e]$\Delta G_{1/2}$; [f]B3LYP/ aug-cc-pVTZ calculations; [g]RCCSD(T)/CBS(TQ5) calculations.

Table S3. Vibrational levels (cm^{-1}) of the $X^1\Sigma^+$ and $a^3\Sigma^+$ states.

$X^1\Sigma^+$						$a^3\Sigma^+$					
υ	This work	υ	This work	υ	This work	υ	This work	υ	This work	υ	This work
0	567.94	27	26616.12	54	43830.66	0	403.16	27	17748.66	54	27895.57
1	1694.78	28	27401.74	55	44271.94	1	1172.25	28	18221.14	55	28099.93
2	2809.56	29	28174.37	56	44699.21	2	1958.28	29	18689.96	56	28289.08
3	3912.20	30	28934.02	57	45112.41	3	2760.63	30	19151.35	57	28463.36
4	5002.66	31	29680.67	58	45511.45	4	3535.02	31	19604.66	58	28622.77
5	6080.89	32	30414.31	59	45896.24	5	4313.52	32	20050.5	59	28767.47
6	7146.83	33	31134.91	60	46266.65	6	5072.13	33	20489.45	60	28897.75
7	8200.44	34	31842.42	61	46622.55	7	5826.47	34	20921.73	61	29014.14
8	9241.65	35	32536.80	62	46963.85	8	6564.09	35	21347.49	62	29117.33
9	10270.43	36	33217.98	63	47290.42	9	7291.26	36	21767.06	63	29207.99
10	11286.71	37	33885.92	64	47602.17	10	8004.75	37	22180.82	64	29286.54
11	12290.45	38	34540.61	65	47898.97	11	8704.92	38	22588.94	65	29353.75
12	13281.62	39	35182.05	66	48180.75	12	9392.93	39	22991.34	66	29410.58
13	14260.17	40	36208.16	67	48447.49	13	10065.76	40	23524.82	67	29457.92
14	15226.06	41	36840.98	68	48699.19	14	10722.1	41	23919.94		
15	16179.25	42	37460.42	69	48935.80	15	11358.44	42	24306.63		
16	17119.72	43	38066.40	70	49157.37	16	11973.24	43	24683.32		
17	18047.42	44	38658.87	71	49364.04	17	12568.3	44	25048.02		
18	18962.32	45	39237.74	72	49555.93	18	13149.22	45	25398.69		
19	19864.39	46	39802.98	73	49733.23	19	13721.7	46	25733.94		
20	20753.61	47	40354.54	74	49896.23	20	14287.39	47	26053.52		
21	21629.94	48	40892.39	75	50045.15	21	14844.21	48	26357.98		
22	22493.36	49	41416.51	76	50180.28	22	15385.89	49	26648.37		
23	23343.85	50	41926.89	77	50301.94	23	15902.22	50	26925.76		
24	24181.38	51	42423.52	78	50410.52	24	16381.96	51	27189.96		
25	25005.94	52	42906.39	79	50506.42	25	16830.82	52	27440.19		
26	25817.52	53	43375.46	80	50590.07	26	17281.74	53	27675.64		

Table S4. Vibrational levels (cm^{-1}) of the $1^5\Delta$, $1^5\Sigma^-$, $d^3\Sigma^+$ and $2^5\Sigma^+$ states.

	$1^5\Delta$	$1^5\Sigma^-$	$d^3\Sigma^+$		$2^5\Sigma^+$	
υ			1st well	2nd well	1st well	2nd well
0	367.59	360.23	1034.82	267.73	351.24	60.85
1	1092.95	1067.99	2840.47	797.98	985.96	173.87
2	1804.73	1754.34	4423.75	1320.84	1722.07	272.76
3	2502.11	2410.24	5947.22		2462.87	357.32
4	3183.88	3028.08	7421.26		3189.41	427.82
5	3847.84	3615.73	8878.22		3902.35	
6	4489.41	4192.62	10359.15		4598.98	
7	5099.06	4758.15	11916.14		5263.96	
8	5657.67	5300.43	13563.76		5858.63	
9	6140.44	5840.54	15301.80		6355.8	
10	6585.96	6407.30	17111.61		6848.76	
11		6988.90	18956.36			

Table S5. Spectroscopic parameters obtained by the icMRCI+Q/56+CV+DK+SO calculations for the 16 Ω states yielded from the $X^1\Sigma^+$, $a^3\Sigma^+$, $d^3\Sigma^+$, $e^3\Sigma^-$, $1^5\Sigma^+$, $2^5\Sigma^+$, and $1^5\Sigma^-$ states.

	T_e /cm^{-1}	R_e /nm	ω_e /cm^{-1}	D_e /eV	Leading Λ-S state compositions near R_e(%)
$X^1\Sigma^+_{0+}$	0.00	0.15923	1138.88	6.3170	$X^1\Sigma^+$ (100.00).
$a^3\Sigma^+_1$	21411.73	0.17200	828.697	3.6842	$a^3\Sigma^+$ (99.99).
$a^3\Sigma^+_{0-}$	21412.60	0.17200	828.711	3.6841	$a^3\Sigma^+$ (100.00).
$d^3\Sigma^+_{0-}$					
**1st well**	28926.54	0.15982	2177.06	3.7098	$d^3\Sigma^+$ (100.00).
**2nd well**	47380.40	0.19867	537.120	1.4218	$d^3\Sigma^+$ (100.00).
$d^3\Sigma^+_1$					
**1st well**	28926.56	0.15982	2177.05	3.7099	$d^3\Sigma^+$ (100.00).
**2nd well**	47380.42	0.19867	537.101	1.4219	$d^3\Sigma^+$ (100.00).
$e^3\Sigma^-_{0+}$	29433.52	0.16968	861.103	4.1088	$e^3\Sigma^-$ (100.00).
$e^3\Sigma^-_1$	29434.40	0.16968	861.092	4.1087	$e^3\Sigma^-$ (99.98).
$1^5\Sigma^+_2$	39169.42	0.20130	483.807	1.4821	$1^5\Sigma^+$ (100.00).
$1^5\Sigma^+_1$	39170.30	0.20128	482.560	1.4873	$1^5\Sigma^+$ (99.94).
$1^5\Sigma^+_{0+}$	39170.52	0.20127	482.204	1.4873	$1^5\Sigma^+$ (99.92).
$2^5\Sigma^+_2$					
**1st well**	50896.83	0.16778	752.828	0.8839	$2^5\Sigma^+$ (99.98).
**2nd well**	57413.69	0.30026	125.508	0.0760	$2^5\Sigma^+$ (98.42), $c^3\Pi$ (1.42), $1^5\Pi$ (0.16).
$2^5\Sigma^+_1$					
**1st well**	50897.26	0.16778	752.841	0.8838	$2^5\Sigma^+$ (100.00).
**2nd well**	57414.34	0.30029	125.288	0.0757	$2^5\Sigma^+$ (98.70), $c^3\Pi$ (0.88), $1^5\Pi$ (0.34).
$2^5\Sigma^+_{0+}$					
**1st well**	50897.55	0.16778	752.860	0.8837	$2^5\Sigma^+$ (100.00).
**2nd well**	57414.56	0.30030	125.206	0.0757	$2^5\Sigma^+$ (98.77), $c^3\Pi$ (0.76), $1^5\Pi$ (0.40).
$1^5\Sigma^-_{0-}$	58859.84	0.17882	724.013	0.8944	$1^5\Sigma^-$ (100.00).
$1^5\Sigma^-_1$	58860.02	0.17882	724.023	0.8944	$1^5\Sigma^-$ (100.00).
$1^5\Sigma^-_2$	58860.90	0.17882	724.024	0.8944	$1^5\Sigma^-$ (99.98).

Table S6. Spectroscopic parameters obtained by the icMRCI+Q/56+CV+DK+SO calculations for the 20 Ω states generated from the $c^3\Pi$, $f^3\Pi$, $1^5\Pi$ and $2^5\Pi$ states.

	T_e/cm^{-1}	R_e/nm	ω_e/cm^{-1}	D_e/eV	Leading Λ-S state compositions near R_e(%)
$c^3\Pi_{0-}$	27253.48	0.16309	969.089	3.8328	$c^3\Pi$ (100.00).
$c^3\Pi_{0+}$	27253.92	0.16309	969.091	3.8328	$c^3\Pi$ (100.00).
$c^3\Pi_1$	27301.11	0.16308	969.436	3.8269	$c^3\Pi$ (99.99), $b^3\Delta$ (0.01).
$c^3\Pi_2$	27348.73	0.16306	969.466	3.8211	$c^3\Pi$ (100.00).
$f^3\Pi_2$	29941.17	0.16491	1065.94	4.0419	$f^3\Pi$ (99.98), $1^5\Pi$ (0.02).
$f^3\Pi_1$	29974.53	0.16475	1001.02	4.0377	$f^3\Pi$ (99.98), $e^3\Sigma^-$ (0.02).
$f^3\Pi_{0+}$	29999.99	0.16361	957.183	4.0345	$f^3\Pi$ (99.98), $e^3\Sigma^-$ (0.02).
$f^3\Pi_{0-}$	30000.03	0.16361	957.225	4.0345	$f^3\Pi$ (99.98), $d^3\Sigma^+$ (0.02).
$1^5\Pi_3$	43593.81	0.19041	615.473	1.7980	$1^5\Pi$ (100.00).
$1^5\Pi_2$	43611.80	0.19041	615.709	1.7957	$1^5\Pi$ (99.98), $1^5\Sigma^+$ (0.02).
$1^5\Pi_1$	43630.02	0.19042	615.884	1.7935	$1^5\Pi$ (99.99), $f^3\Pi$ (0.01).
$1^5\Pi_{0+}$	43648.33	0.19042	615.901	1.7912	$1^5\Pi$ (100.00).
$1^5\Pi_{0-}$	43648.46	0.19042	615.942	1.7912	$1^5\Pi$ (99.99), $e^3\Sigma^-$ (0.01).
$1^5\Pi_{-1}$	43667.11	0.19042	615.965	1.7890	$1^5\Pi$ (100.00).
$2^5\Pi_{-1}$	52965.37	0.18881	765.702	1.1869	$2^5\Pi$ (100.00).
$2^5\Pi_{0-}$	52987.54	0.18880	765.943	1.1841	$2^5\Pi$ (100.00).
$2^5\Pi_{0+}$	52987.76	0.18880	765.999	1.1841	$2^5\Pi$ (100.00).
$2^5\Pi_1$	53009.71	0.18879	766.254	1.1814	$2^5\Pi$ (100.00).
$2^5\Pi_2$	53031.87	0.18878	766.512	1.1786	$2^5\Pi$ (100.00).
$2^5\Pi_3$	53054.04	0.18878	766.742	1.1758	$2^5\Pi$ (100.00).

Table S7. Spectroscopic parameters determined by the icMRCI+Q/56+CV+DK+SO calculations for the 9 Ω states generated from the $b^3\Delta$ and $1^5\Delta$ states.

	T_e/cm^{-1}	R_e/nm	ω_e/cm^{-1}	D_e/eV	Leading Λ-S state compositions near R_e(%)
$b^3\Delta_3$	26278.36	0.17073	854.053	3.9602	$b^3\Delta$ (99.98), $c^3\Pi$ (0.02).
$b^3\Delta_2$	26279.89	0.17072	854.068	3.9602	$b^3\Delta$ (99.96), $c^3\Pi$ (0.04).
$b^3\Delta_1$	26281.21	0.17071	853.871	3.9600	$b^3\Delta$ (99.98), $c^3\Pi$ (0.02).
$1^5\Delta_{0-}$	55585.46	0.17621	738.107	0.8303	$1^5\Delta$ (100.00).
$1^5\Delta_{0+}$	55585.48	0.17621	738.054	0.8303	$1^5\Delta$ (100.00).
$1^5\Delta_1$	55587.22	0.17620	738.327	0.8303	$1^5\Delta$ (100.00).
$1^5\Delta_2$	55587.88	0.17620	738.407	0.8303	$1^5\Delta$ (100.00).
$1^5\Delta_3$	55588.53	0.17620	738.478	0.8303	$1^5\Delta$ (100.00).
$1^5\Delta_4$	55590.51	0.17619	738.698	0.8304	$1^5\Delta$ (100.00).
